# Hydrothermal chimneys host habitat-specific microbial communities: analogues for studying the possible impact of mining seafloor massive sulfide deposits

**DOI:** 10.1038/s41598-018-28613-5

**Published:** 2018-07-10

**Authors:** Yuchen Han, Giorgio Gonnella, Nicole Adam, Axel Schippers, Lia Burkhardt, Stefan Kurtz, Ulrich Schwarz-Schampera, Henrike Franke, Mirjam Perner

**Affiliations:** 1 Universität Hamburg, MIN-Fakultät, Biocenter Klein Flottbek, Molecular Biology of Microbial Consortia, Ohnhorststr. 18, 22609 Hamburg, Germany; 2 Universität Hamburg, MIN-Fakultät, ZBH - Center for Bioinformatics, Bundesstraße 43, 20146 Hamburg, Germany; 3 Federal Institute for Geosciences and Natural Resources (BGR), Stilleweg 2, 30655 Hannover, Germany; 4 Heinrich-Pette-Institut, Leibniz-Institute for Experimental Virology, Martinistraße 52, 20251 Hamburg, Germany; 5 Present Address: GEOMAR Helmholtz Center for Ocean Research Kiel, Geomicrobiology, Wischhofstr. 1-3, 24148 Kiel, Germany

## Abstract

To assess the risk that mining of seafloor massive sulfides (SMS) from extinct hydrothermal vent environments has for changing the ecosystem irreversibly, we sampled SMS analogous habitats from the Kairei and the Pelagia vent fields along the Indian Ridge. In total 19.8 million 16S rRNA tags from 14 different sites were analyzed and the microbial communities were compared with each other and with publicly available data sets from other marine environments. The chimneys appear to provide habitats for microorganisms that are not found or only detectable in very low numbers in other marine habitats. The chimneys also host rare organisms and may function as a vital part of the ocean’s seed bank. Many of the reads from active and inactive chimney samples were clustered into OTUs, with low or no resemblance to known species. Since we are unaware of the chemical reactions catalyzed by these unknown organisms, the impact of this diversity loss and bio-geo-coupling is hard to predict. Given that chimney structures can be considered SMS analogues, removal of sulfide deposits from the seafloor in the Kairei and Pelagia fields will most likely alter microbial compositions and affect element cycling in the benthic regions and probably beyond.

## Introduction

The ocean seafloor and subsurface host a tremendous reservoir for seafloor massive sulfides (SMS). SMS are created through hydrothermal convection and fluid circulation. They commonly have a high base metal and sulfide content. Particularly active fluid discharges from hydrothermal chimneys are striking structures on the seafloor. Along the thermal and chemical gradients in SMS environments a phylogenetically diverse suite of piezophilic to thermophilic and aerobic to anaerobic microorganisms, with versatile metabolic strategies exist: metabolisms include the ability to use numerous inorganic energy sources (e.g., massive sulfides, ferrous iron, H_2_S, H_2_), and to fix CO_2_ autotrophically^1–8^. After hydrothermal activity ceases, the chimney structures continue to react with the ambient low temperature seawater. Inactive hydrothermal chimneys also provide a special habitat where oxidation of metal sulfides marks a significant source for chemosynthetic microorganisms^9,10^ and appear to be driven to a certain extent by mineralogy^11^. Given that the chimney habitat itself selects for the community composition but microbial processes in turn also contribute to the formation and conversion of the chimney mineralogical composition, a strong tie between microbe-mineral interactions and biogeochemical cycling is obvious.

In the near future mining of SMS may become economically feasible since mining techniques and extraction technology have advanced and commodity prices and metal demand have increased – due to the accelerating industrialization of countries like China and India^12^. SMS potentially worth mining cover extensive areas in and mostly under the seafloor surface and thus can be difficult to find and sample. In contrast, hydrothermally derived chimney structures are relatively easily accessible sulfide deposits which represent cross sections of SMS habitats found under the seafloor^12,13^. Therefore, chimneys can be considered as SMS analogues and used to study the impact SMS mining has on microbial diversity and the biogeochemical cycling in the entire area. From the technical point of view, it is hard to develop mining equipment suitable for low pH and high temperature as encountered in hydrothermally active regions, thus hydrothermally extinct areas are considered for mining. However, while active chimneys can be regenerated in weeks to years, SMS in hydrothermally extinct fields take tens of thousands of years to concentrate economically interesting metals in large amounts, indicating that the deposits are not renewable resources^14^.

Many environmental impacts of deep-sea mining have been predicted, such as changes in water quality, sedimentation, endemic fauna and microbial communities^12,15–18^. SMS mining activities will produce tons of drifting particles, which might clog and contaminate nearby environments and interfere with environmental processes^19^. Also, mining activities cause mixing of solid with liquid components on the sea floor and change essential nutrients and water properties in the deep-sea^19^. With only a few of the possible effects pointed out here, deep-sea mining of SMS deposits may result in the loss of ecologically important habitats, change the local benthic community and destroy the region’s ability to sustain itself. To minimize the risk of environmental destruction and to maintain the sustainability of marine environments, it is very important to design suitable management strategies for deep-sea mining.

In 2015 the International Seabed Authority (ISA) assigned a license to the German Federal Institute for Geosciences and Natural Resources (BGR) to explore deep-sea hydrothermal vents in a specific area of the southwestern Indian Ocean, which is enriched in massive polymetallic sulfides. The work presented here includes sites located in the Kairei field and the newly found Pelagia vent field. The Kairei hydrothermal field was first discovered in 2001 along the Central Indian Ridge (CIR)^20,21^. Since then, a geochemical and geological^20,22,23^ as well as a biological^9,21,24,25^ background has been established. The Kairei chimneys contain massive sulfide precipitates^22,26^ and mafic to ultramafic wall rocks are discussed to represent the metal source. The major mineral components of inactive chimney structures from Kairei are chalcopyrite (CuFeS_2_) and pyrrhotite (Fe_1−x_S)^9^, which previously were precipitated from metal-rich hydrothermal vent fluids^26^. The emitted fluids have a low pH and are enriched in H_2_S and H_2_, but hold low methane concentrations^27^. Some studies on the microbial communities from inactive chimneys^9,11^ as well as hydrothermal fluids and active chimneys^24,25^ already exist for the Kairei vent field. Their microbial communities have also been compared to those from other chimney habitats, demonstrating diverse microbial communities in active chimneys and show that mineralogy is an important factor for shaping inactive chimney microbial community compositions^11,25^. However, differences and similarities between communities from Kairei active and inactive chimneys were not assessed. While the Kairei area was already studied for more than a decade, the Pelagia vent field at the Southeast Indian Ridge was newly discovered during the cruise INDEX2014 (BGR, pers. comm.) and this is the first report on its microbial and mineralogical composition. The aim of this study was to identify whether active venting and inactive chimney structures in the Kairei and Pelagia areas host habitat specific bacterial and archaeal communities. Since hydrothermally formed chimneys can be considered as windows into SMS areas, targeted for mining, the knowledge of the specificity of chimney communities can give insights into the possible “diversity loss”, if removed through mining from the seafloor. This can help in assessing the role of altered biogeochemical cycling for the benthic habitat.

## Results and Discussion

The aim of this study was to identify taxa that appear to be highly specific for chimney structures as analogues for SMS to assess the potential diversity loss if mining of comparable structures would take place. For this purpose we assessed the microbial communities of fourteen rock, fluid and water samples from the Kairei and Pelagia venting areas and nearby in the Indian Ocean. Mineralogy of the chimney structures was described and linked to the presence of taxa. OTUs (Operational Taxonomic Units) specific to a habitat type were identified. Using beta diversity analyses we investigated the similarities and dissimilarities among the samples in the Kairei and Pelagia environments and illustrated their relation to samples from comparable biotopes available from the literature.

### Mineralogical and geochemical relations of hydrothermal precipitates

From the sulfide precipitates collected during the INDEX2016 cruise seven active and inactive chimney samples were selected as containing two types of mineralization: i) three copper-rich chimney edifices and fragments and ii) four beehive-shaped iron-zinc-rich chimneys (see Table 1).

**Table 1:** Semi-quantitative distribution of sulfide minerals and main element compositions of hydrothermal precipitates from the Kairei and Pelagia hydrothermal vent fields.

Sample		Semi-quantitative mineral composition	Quantitative main element composition	Description
**Kairei area**				
INDEX2016-06ROV03-A	S1	ccp+++ py/mrc+ sp++	Cu: 12.6–25.8 wt.% Fe: 18.5–25.6 wt.% Zn: 10.3–29.7 wt.%	fragment of inactive chimney, Kairei active zone
INDEX2016-06ROV03-N	S2	ccp 0 py/mrc+++ sp+++ iso 0	Cu: 0.3–0.4 wt.% Fe: 23.8–28.3 wt.% Zn: 28.9–31.7 wt.%	inactive beehive shaped chimney, Kairei inactive zone
INDEX2016-12ROV06-E	S7	ccp +++ py/mrc+ sp++	Cu: 18.1–26.2 wt.% Fe: 21.2–28.4 wt.% Zn: 6.8–24.3 wt.%	inactive chimney edifice, Kairei active zone
INDEX2016-12ROV06-F	S8	ccp+++ py/mrc+ sp++ anh 0	Cu: 25.3–26.5 wt.% Fe: 27.1–29.0 wt.% Zn: 6.5–10.7 wt.%	fragment of active chimney, Kairei HF
**Pelagia area**				
INDEX2016-16ROV08-E	S5	ccp+ py/mrc+++ sp +	Cu: 1.3–7.4 wt.% Fe: 33.3–44.4 wt.% Zn: 0.9–12.2 wt.%	active beehive shaped chimney, Pelagia HF
INDEX2016-20ROV10-G	S6	ccp 0 py/mrc+++ po+ sp++ iso +	Cu: 1.5–1.7 wt.% Fe: 41.5–42.2 wt.% Zn: 7.0–7.1 wt.%	fragment of active chimney, Pelagia HF
INDEX2016-20ROV10-J	S4	ccp 0 py/mrc+++ sp+	Cu: 0.1–0.8 wt.% Fe: 29.9–36.2 wt.% Zn: 0.5–3.3 wt.%	fragment of beehive shaped inactive chimney, Pelagia HF

Geochemical compositions are given in min.-max. values from interior to outer part of the sample. Mineral abbreviations: ccp = chalcoyprite (CuFeS_2_), py/mrc = pyrite/marcasite (FeS_2_), sp = sphalerite (ZnS), po = pyrrhotite (Fe_1−x_S), iso = isocubanite (CuFe_2_S_3_), anh = anhydrite (CaSO_4_) +++ abundant, ++ common, + traces, 0 acessory. HF = hydrothermal field.

Copper-rich chimney edifices and fragments (S1, S7, S8) sampled from the Kairei hydrothermal field are dominated by chalcopyrite, with minor pyrite, marcasite, wurtzite and sphalerite. Conduits are lined by massive chalcopyrite coexisting with granular wurtzite and pyrite/marcasite. Wurtzite grains often occur within the massive chalcopyrite aggregates, with zonal enrichment of secondary chalcopyrite inclusions attesting to replacement processes forming so-called chalcopyrite diseases textures^28^. The occurrence and grain size of granular chalcopyrite decrease towards the outer parts of the chimneys. Simultaneously an increasing amount of disseminated sphalerite coexisting with pyrite and minor chalcopyrite can be observed in the outer parts. This indicates decreasing temperatures towards the outer parts of the chimney due to changes in the precipitation of chalcopyrite and sphalerite. Active chimney edifices and fragments contain accessory, lath-shaped and fibrous relics of anhydrite filling interstices and occasionally inactive conduits. The absence of anhydrite in inactive chimney edifices (S1) attests to anhydrite solubility in seawater during cooling. All chimney walls are coated with iron-oxyhydroxide due to seawater oxidation.

Iron-zinc-rich beehive chimneys were sampled at the Kairei (S2) and the Pelagia (S4, S5, S6) hydrothermal field. They contain complex intergrowth of different minerals and mineral textures, with large amounts of homogeneously distributed pore spaces that allow fluid flow towards the chimney walls^29^. The high porosity derives from aggregates of collomorph pyrite/marcasite, sphalerite surrounded by chalcopyrite, lath-shaped pyrrhotite and amorphous silica. Chimney interiors are characterized by traces of sulfides formed from high-temperature precipitation such as chalcopyrite-isocubanite surrounded by pyrite and filling conduits. Outer portions of the chimneys consist of lower-temperature precipitates such as sphalerite, pyrite/marcasite and locally amorphous silica. Active chimneys are nearly fresh and weathering products can only be observed on the outer wall in contact with seawater. However, inactive chimney edifices (S4) show starting weathering processes reflected by replacement of chalcopyrite-isocubanite by secondary copper minerals (e.g. covellite).

Active as well as inactive chimney edifices, are characterized by distinct compositional zoning from the core towards the rim reflecting strong gradients in fluid temperature and composition^30,31^. Mineral and geochemical compositions of copper-rich chimney edifices (S1, S7, S8) indicate formation conditions at temperatures at or above 300 °C. Specific fluid compositions cause higher amounts of high-temperature precipitates than in iron-zinc-rich beehive chimneys (S2, S4, S5, S6).

### Bacterial and archaeal communities of the Kairei and Pelagia areas

Kairei and Pelagia microbial communities from three active chimneys and four inactive chimneys (for Archaea three inactive chimneys) were compared with two hydrothermal fluids, one sulfide talus sample, two plume water samples and two open ocean samples (Table 2). Our sequencing yielded 10.3 million bacterial and 9.6 million archaeal 16S rRNA amplicons, which resulted in 140785 and 121333, respectively, OTUs (at a dissimilarity of 3%) (Table 2, and Supplementary Table S1). Total cell counts of the fluids were between 1.3 × 10^4^ ± 1.6 × 10^3^ cells ml^−1^ and 1.2 × 10^5^ ± 5.7 × 10^4^ cells ml^−1^ (Table 2). Rarefaction curves of the sequenced samples demonstrate a similar good coverage of both the bacterial and archaeal communities (Supplementary Fig. S1). In our analyses, we applied an open-reference OTU picking procedure^32^, which first collects sequences into OTUs by similarity to database sequences (here termed reference-based OTUs) and clusters the remaining sequences into OTUs by their similarity to each other (here termed *de novo* OTUs). Most OTUs in both the bacterial (96%) and archaeal (99%) communities were *de novo* OTUs, although sequence counts were lower than those of reference-based OTUs, i.e. they consist of 31% and 34%, of the total bacterial and archaeal 16S tags, respectively (Supplementary Table S1).

**Table 2:** Total cell counts and number of analyzed 16S rRNA genes.

Sample name	Sample name	Location and sample types	Cell number per ml	Number of 16S rRNA sequences	
				bacteria	archaea
**open ocean sample**					
INDEX2016-01PS	RF1	sea water, depth 2180 m, approx. 10 km away from Kairei venting site	1.62E +04 ± 3.61E+03	631890	766553
INDEX2016-15PS	RF4	sea water, depth 3566 m; approx. 10 km away from Pelagia venting site	1.30E +04 ± 1.56E+03	679918	641362
**Kairei area**					
INDEX2016-09PS	F5	water sample from just above the plume, 2327 m depth	1.50E +04 ± 1.80E+03	815297	879790
INDEX2016-06ROV-03-KIPS-B	F1	low temperature hydrothermal diffuse fluids (23 °C)	1.23E +05 ± 5.74E+04	757618	940285
INDEX2016-12ROV06-F	S8	active chimney	no data	542480	315288
INDEX2016-06ROV03-A	S1	inactive chimney, Kairei active zone	no data	920258	1140926
INDEX2016-06ROV-03-N	S2	inactive chimney, Kairei inactive zone	no data	619471	no data
INDEX2016-12ROV06-E	S7	inactive chimney, Kairei active zone	no data	575095	504492
**Pelagia area**					
INDEX2016-17PS plume max	F9	plume water sample, 3643 m depth	1.38E +04 ± 8.01E+03	897847	1044117
INDEX2016-16ROV08-B	S3	sulfide talus	no data	921885	853219
INDEX2016-16ROV08-KIPS-B	F6	hydrothermal diffuse fluid (153 °C)	3.89E +04 ± 4.29E+03	980269	966748
INDEX2016-16ROV08-E	S5	active chimney	no data	637597	420015
INDEX2016-20ROV10-G	S6	active chimney	no data	600895	422585
INDEX2016-20ROV10-J	S4	inactive chimney	no data	717195	697248
TOTAL				10297715	9592628

The results of the taxonomic assignments of the bacterial and archaeal OTUs identified in the different habitats are presented as plots in the supplementary results, summarized as phyla/class levels (Supplementary Fig. S2a-f), orders (Supplementary Fig. S3a-c) and genera (Supplementary Fig. S4a-f). Furthermore, to gain a better understanding of the OTUs that are of relevance for a given habitat we listed those that were found in our analyses exclusively (Supplementary Tables S2, S3 and S4) or were significantly more abundant in one habitat type (Supplementary Tables S5). As we have shown in a previous study^33^ apparent habitat endemicity in 16S analyses of the microbial communities for marine environments is in many cases likely to be an artifact of an insufficient coverage so that individuals of very rare OTUs are not detected. Nevertheless, the absence in a habitat of an OTU detected in another habitat, is an indication of its rareness. Hence, we present the results of both analyses together and refer in the following text to apparently “exclusive” OTUs, as well as OTUs significantly more abundant in a habitat type, as *habitat specific OTUs*. We present, in the following sections, three comparisons of habitat types: (i) active versus inactive chimneys, (ii) inactive chimneys versus all other samples and (iii) water samples versus all chimneys samples.

#### Active chimneys versus inactive chimneys

Our analyses showed a total of 29142 OTUs (13163 and 15979, respectively, in bacterial and archaeal analyses) detected in at least one of the active chimneys but not in the inactive chimneys (Supplementary Table S2). Among these active chimney specific OTUs the most abundant ones were classified *Epsilonproteobacteria* in particular with mesophilic *Campylobacterales* and thermophilic *Nautiliales* as well as thermophilic *Aquificae*. OTUs significantly more abundant in active chimneys than in inactive chimneys were classified as thermophilic Archaea: *Archaeoglobales* (sulfate-reducing Archaea)^34^, *Thermococcales* (reducing sulfur to H_2_S)^35^, *Thermoprotei* (obtaining energy by oxidation of H_2_, H_2_S, and elemental sulfur)^36^ and *Methanococcales* (forming methane by CO_2_ reduction with H_2_)^37^ (Supplementary Tables S2,S5). The Bacteria and Archaea found in the Kairei and Pelagia active chimneys are common for this type of habitat^1,38–43^. They are ideally adapted to the thermal and chemical gradients hallmarking the chimney conduit. The primary producers but also heterotrophs can make use of the readily available reduced compounds provided by the hot, reduced hydrothermal fluids^4,44^. *Sulfurimonas* species, one of the abundant epsilonproteobacterial species in active chimneys, can strongly influence their habitats through autotrophic denitrification coupled with oxidation of sulfides or metal sulfide to soluble metal sulfate^45^, e.g. remediation of metal sulfide from contaminated sediments^46^ and remediation of nitrate contamination from groundwater^47^. Many species in the order *Nautiliales* and *Aquificae* can reduce element sulfur to produce sulfide^4,44,48^, resulting in the formation of metal sulfides in their habitats. Remarkably, most OTUs among active chimney specific Archaea could not be taxonomically classified (besides kingdom level), due to lacking similarity to the sequences in the reference databases (Supplementary Table S2). Detecting high proportions of previously unknown sequences is not uncommon in such environments^38,43^: we remain unaware of the role these microbes have for biogeochemical cycling, demonstrating our still quite limited understanding of these settings.

81002 OTUs (45639 and 35363, respectively, from bacterial and archaeal analyses) were present in at least one of the inactive chimney samples, but not found in active chimneys. Among the most abundant bacterial OTUs we found phylogenetically distinct groups (Supplementary Table S2): *Desulfobulbus*, and SAR324 (*Deltaproteobacteria*), *Oceanospirillales*, *Alteromonadales*, *Chromatiales* (*Nitrosoccus* and *Acidiferrobacter*), and *Thiotrichales*. The archaeal OTUs were classified as Woesearchaeota (DHVEG-6, deep-sea hydrothermal vent euryarchaeota group 6), *Thaumarchaeota*, in particular *Nitrosopumilus*, and *Thermoplasmata* (Marine Group III). Cultured representatives of these microorganisms catalyze ammonia-oxidation^49–51^, sulfide- and/or hydrogen-oxidation^52–54^, can grow autotrophically with iron-sulfide minerals^55^ and are involved in iron- and sulfur-cycles^56–58^. Affiliates of the *Chromatiales* can use sulfide minerals such as pyrite as energy source^59^, while the members of *Desulfobacterales* can produce extracellular pyrite via reduction of sulfate^60,61^, indicating that these species can also influence the mineral composition in their habitats. *Nitrosopumilius* is an autotrophic ammonia-oxidizing marine archaeon^51^, which can use very limited amounts of nitrogen and carbon to maintain life^62^. Affiliates of this group have been found in other inactive vents^38,63^, too, suggesting that its metabolism may play a significant role for nitrogen and carbon cycling in such chimneys^62^. Even though some *Nitrosopumilius* related sequences were also found in active chimneys, the abundance was much lower than in the inactive chimneys in the same field^38^. The trend of high abundance of *Nitrosopumilius* related sequences in inactive chimneys coincides with our findings. In summary, the distinct microbial patterns of the active versus the inactive chimneys confirm that the different chimney environments select for the specific microbial compositions.

#### Inactive chimneys versus all other samples

Some of the inactive chimney OTUs discussed in the previous section, could be absent in active chimneys, but still be present in other kind of habitats. Our results show that among the 81002 OTUs present in inactive chimneys but not in active chimneys (see previous section), 70625 OTUs were also absent from water, plume and hydrothermal fluid samples (39467 from the bacterial, 31158 from archaeal analysis). The most abundant of these OTUs were classified as *Desulfobulbus* and members of the *Thiotrichales* (Supplementary Table S3). Other inactive chimney specific OTUs were scattered across a phylogenetically broad group of taxa including *Alpha*-, *Gamma*-, *Deltaproteobacteria*, *Bacteroidetes*, *Planctomycetes*, *Actinobacteria*, *Acidobacteria*, *Chloroflexi* and DHVEG-6, Marine Benthic group E and unassigned Archaea (Supplementary Table S3). The Marine Benthic group E is frequently detected in deep-sea sediments^64^ and inactive chimneys^9^ and placed within the *Euryarchaeota*. Their phylogenetic position between members of the *Methanobacteriales* and the *Methanosarcinales* may be indicative of a possible methanogenic phenotype^65^. Similar taxonomic patterns have also been found in inactive chimneys before^11,38,43^. Thus, inactive chimneys support a high microbial diversity and the habitat type may even function as one of the homes to the oceanic seed library. Likely, the stability of the inactive chimney environment, i.e. ambient temperatures and slow chemical reaction processes, both weathering but also microbially catalyzed redox reactions, as well as the food sources provided by the chimney mineralogical composition provide an attractive habitat for a broad range of microorganisms.

#### Open ocean versus hydrothermal samples

16153 bacterial and 22094 archaeal OTUs were found in at least one of the open ocean samples (RF1 and RF4) but not in any of the chimney samples (Supplementary Table S4). Among OTUs detected in both habitats, several significant differences were also noted (Supplementary Tables S4 and S5): some of the major differences were related to water samples containing larger proportions of heterotrophic *Oceanospirillales* and *Alteromonadales* (*Gammaproteobacteria*) and SAR324 (*Deltaproteobacteria*). *Oceanospirillales* and *Alteromonadales* have been repetitively found in ocean water samples^58,66–69^. Versatile metabolisms (lithotrophy and heterotrophy) for SAR324 make these organisms ubiquitous in the global marine biosphere^58^, as in water samples^58^ (and this study), inactive chimneys (this study), hydrothermal plume^58,70^ and sediments^71^. Members of the *Thaumarchaeota* Marine group I, which are typically associated with oxic marine coastal waters^72,73^, dominated much of the archaeal communities in water samples. Phototrophic *Cyanobacteria* and affiliates of the SAR11 clade and SAR116 clade (*Alphaproteobacteria*) have been found in water samples as well^74,75^ and were also detected in our water samples. These species can perform photosynthesis and consume organic carbons, while most dominant species in hydrothermal fields are chemolithotrophs, indicating that the different environmental conditions between open oceans and hydrothermal vents select the species according to their physiological characteristics and result in different diversities among these habitats.

### Linking mineralogy with the abundance of Bacteria and Archaea

Our tested active and inactive chimneys in both fields are rich in two or more metal sulfides (Table 1). The formation of these minerals and degree of rock alteration depend on the temperature and the chemical composition of hydrothermal fluids as well as the age of chimneys, respectively^76^, explaining the distinct mineralogical composition in the tested chimneys (Table 1). To examine whether the mineral compositions influence the bacterial and archaeal diversities, the proportions of chalcopyrite, pyrite and sphalerite relative to the microbial community compositions were analyzed (Supplementary Table S6). Generally, no significant difference between the appearance of some of the dominant microbes and mineral compositions could be established (Supplementary Table S6). Consequently, metal contents (alone) do not appear to play a primary role for microbial community compositions. However, based on the inferred metabolisms from the identified microbes the availability of distinct sulfur species appears to be relevant: *Sulfurimonas*, *Sulfurovum* and *Nitratifractor* use reduced sulfur compounds as electron donors^4,45^, *Desulfobulbus* and *Archaeoglobi* are sulfate-reducers^34,57,61^, and *Thermoplasmata* can reduce sulfur to sulfide^77^. Hence, besides temperature, the type of sulfur species appear to be one major environmental selective factor for shaping the microbial chimney communities. This is also supported by previous findings where different microbial communities and mineral compositions from inactive chimneys in the Iheya North where compared to those from the Kairei fields^9^. Major minerals in Iheya North are sulfate minerals (barite), while the Kairei field is still nascent and composed of reduced sulfide minerals (chalcopyrite)^9^. The reduced status of sulfur in the Kairei field appears to result in a higher diversity of microbial communities than in the Iheya North samples^9^.

### Specificity and relatedness of the different Kairei and Pelagia communities

**Figure 1:**
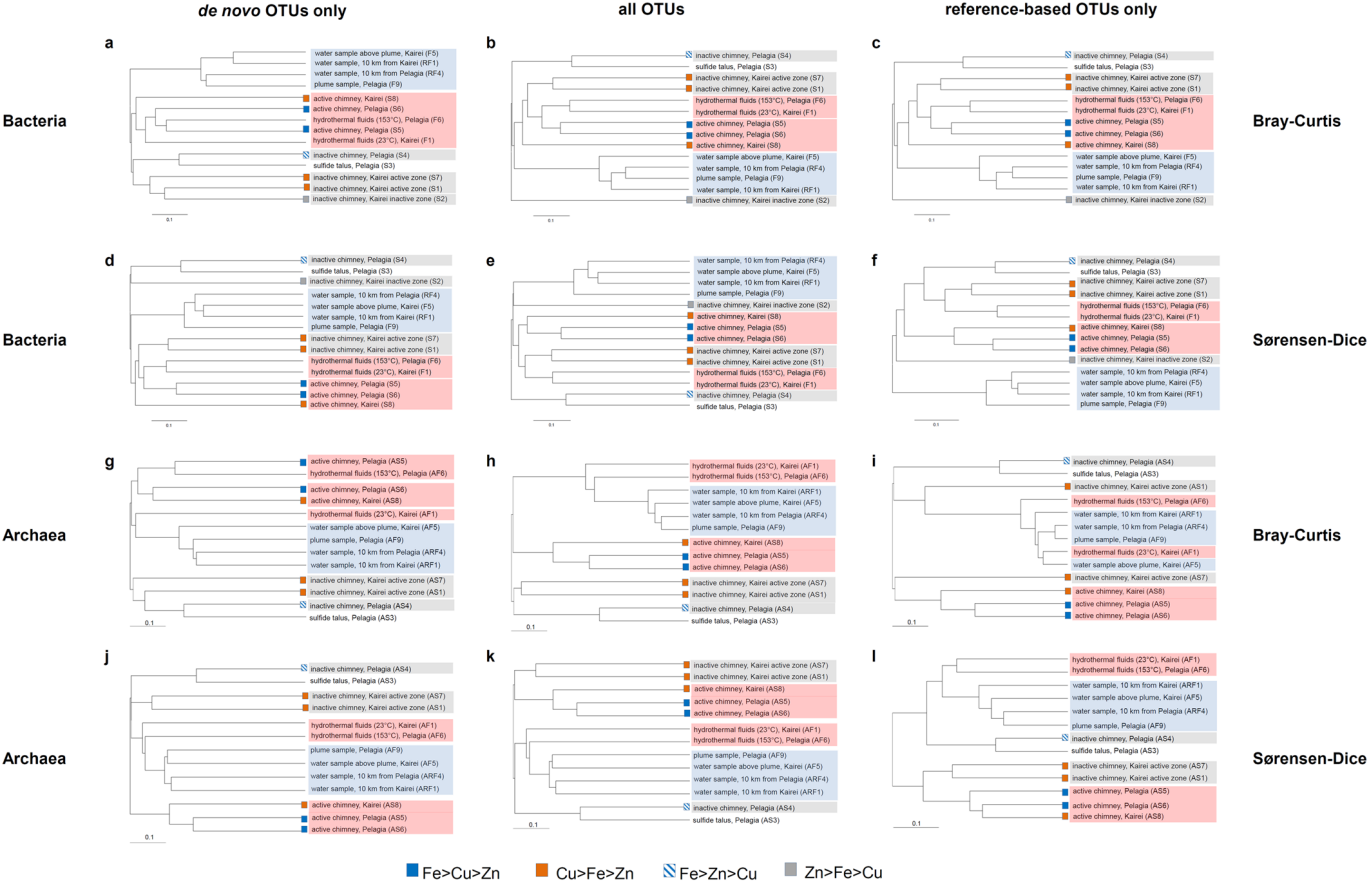
UPGMA clustering trees of fourteen marine samples from the Kairei and Pelagia area. Calculations were performed for bacteria (**a**–**f**) and archaea (**g**–**l**) using the Bray-Curtis (**a**–**c**,**g**–**i**) index or the Sørensen-Dice (**d**–**f**,**j**–**l**) index. Only *de novo* OTUs (**a**,**d**,**g**,**j**), all OTUs (reference-based and *de novo*, **b**,**e**,**h**,**k**) and only reference-based OTUs (**c**,**f**,**i**,**l**) were considered. The sample names are boxed in red for warm/hot fluids and active venting chimneys, in grey for inactive chimneys and blue for water column samples. The color coding of the squares denote the dominant element compositions in the minerals. For details on mineralogy see Table 1.

The UPGMA clustering of the samples, based on beta diversity analyses, yielded quite different results depending on whether *de novo* and/or reference-based OTUs were considered and whether the Sørensen-Dice or Bray-Curtis index was used for analyses (Fig. 1). In all analyses water samples always clustered as one group indicative of these samples being clearly different to all the other investigated habitats. Interestingly enough, only analyses of Bacteria (if sequence counts were considered) (Fig. 1a) and of Archaea (regardless of whether sequence counts were considered or not) (Fig. 1g,j) led to clustering of active versus inactive (including sulfide talus) chimney communities if sequences of *de novo* OTUs were used. It is the same case for Archaea if sequences of all OTUs were used and sequence counts were considered (Fig. 1h), but not if computed using only sequences of reference-based OTUs (Fig. 1i,l). Consequently, the *de novo* OTUs, for which we lack knowledge of possible metabolisms and bio-geo coupling processes, and which in some cases made up a large proportions of the community (up to 99%), have a profound effect on segregating for a habitat specific community.

**Figure 2:**
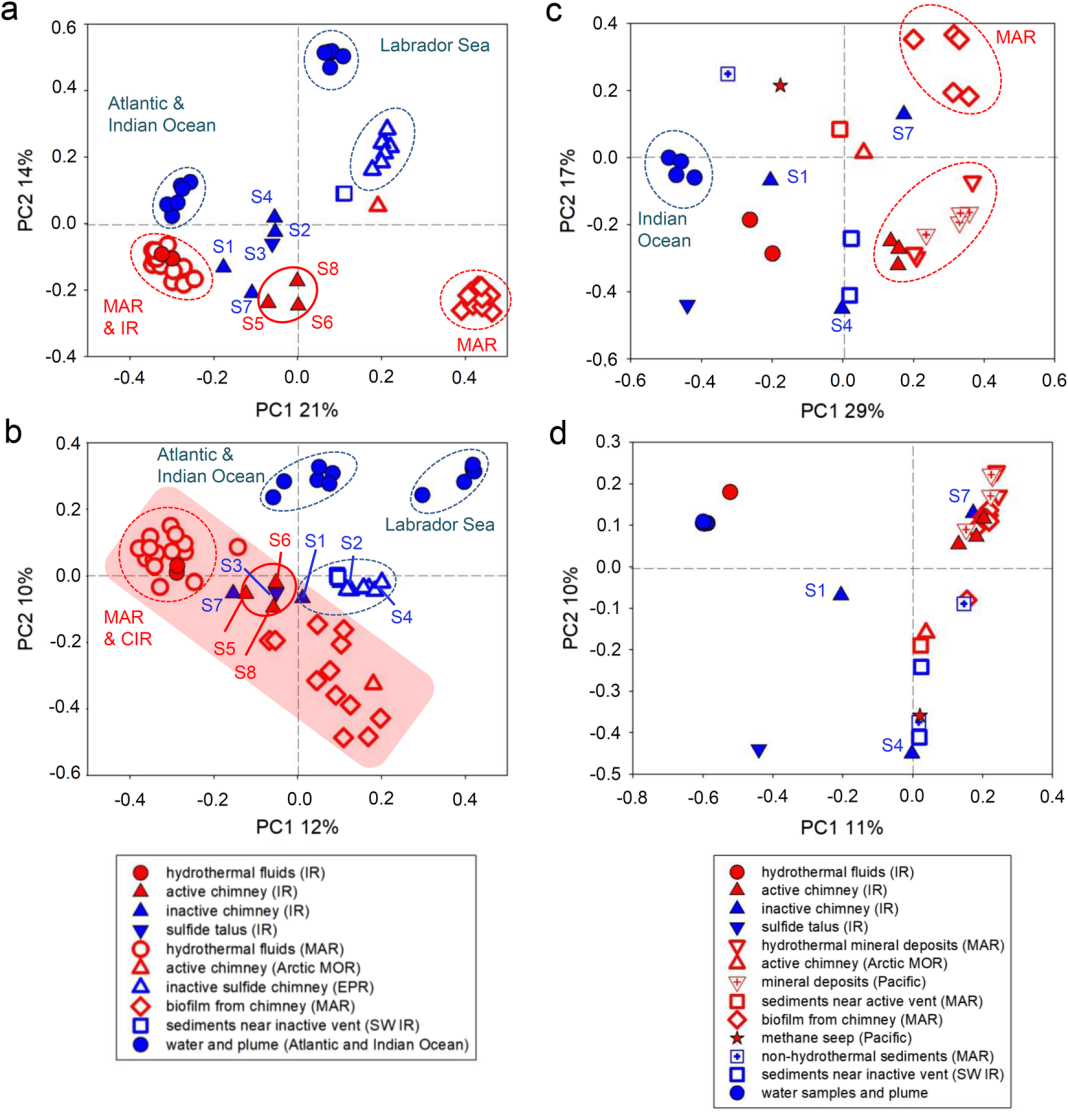
PCoA analysis performed on samples from different marine environments. (**a**,**c**) Sørensen-Dice and (**b**,**d**) Bray-Curtis indices and reference-based OTUs for Bacteria (**a**,**b**) and Archaea (**c**,**d**) were used. The symbols correspond to the different bacterial and archaeal communities from inactive and actively venting chimneys, hydrothermal fluids, and water samples. Communities from this study, i.e. from the Kairei and Pelagia areas along the Indian Ridge (IR), are named hydrothermal fluids (IR), sulfide talus (IR), active chimney (IR), inactive chimney (IR) as well as some of the water and plume samples (for details see Table 1). Additionally, chimney biofilms from the Rainbow and Lucky Strike vents^79^ and from hydrothermal fluids along the Mid-Atlantic Ridge (MAR)^33^, sediments from an inactive hydrothermal vent region in the southwest region of the Indian Ridge (SWIR)^84^, inactive sulfide chimneys from the East Pacific Rise (EPR)^10^, an actively venting chimney from the Arctic mid-ocean ridge (MOR)^80^, mineral deposits from the MAR and the Pacific^81^, sediments near an active vent on the MAR^82^, sediments from the bathypelagic near the MAR^82^, methane seep^83^ and open ocean samples from the Atlantic and Labrador Sea^33,78^ were used for the analyses. The red area indicates the clustering of primarily hydrothermally influenced communities. The first two principal coordinates (PC1, and PC2) for the subsamples of the environments are plotted. The axes are labeled by the percent variation explained by the PCs. Dotted circles represent the sub-clusters of communities from similar types of environments.

To place our samples into context with communities inhabiting other marine habitats, we assessed a total of 37,038,638 bacterial and 6,564,461 archaeal 16S rRNA amplicons. Hereby we considered only reference-based OTUs (in order to allow comparison of studies with results from different regions of the 16S rRNA gene) taxonomically assigned to Bacteria or Archaea, respectively (in order to include results from studies using universal primers). These sequences were from open ocean samples in the Atlantic^33^, the Labrador Sea^78^, hydrothermal fluids and biofilms from chimneys along the Mid-Atlantic Ridge (MAR)^33,79^, an active venting chimney from the Arctic mid-ocean ridge (MOR)^80^, mineral deposits from the MAR and the Pacific^81^, inactive chimneys from the East Pacific Rise (EPR)^10^, sediments near an active vent on the MAR^82^, sediments from the bathypelagic near the MAR^82^, methane seep^83^ and sediments from an inactive hydrothermal venting site on the southwest Indian ridge (SWIR)^84^. PCo analyses showed that bacterial communities from the Kairei and Pelagia chimneys were distinctly different to those from other MOR samples (Fig. 2a). When taking sequence abundance into account, there is a general trend of all analyzed bacterial communities from hydrothermally influenced environments to form one distinct cluster while those of the non-hydrothermally influenced samples are scattered outside of this group (Fig. 2b). However, the inactive chimneys S1 and S7 and to a lesser extent the sulfide talus S3 do not follow this tendency but actually cluster with bacteria from active venting regimes. Given that inactive chimneys S1 and S7 were sampled from active venting zones and the sulfide talus S3 was collected in a diffuse venting area this is not too surprising. Bacterial communities of the inactive chimneys S2 and S4 from the inactive zone, cluster together with those from inactive sulfide chimneys from the EPR and a sediment sample from an inactive venting region on the SWIR (Fig. 2b). According to the Sørensen-Dice index, the archaeal communities of inactive chimneys and the sulfide talus sample appeared scattered but isolated from the other mineral deposits and hydrothermally influenced samples (Fig. 2c). Active chimneys though clustered with other active venting deposits from geographically distinct locations (Fig. 2c). When quantitative measures were applied only S1 was isolated while S4 grouped with non-hydrothermal samples and S7 clustered with hydrothermally influenced habitats (Fig. 2d). It appears that different parameters are shaping bacterial and archaeal communities.

### Rare microbial taxa and unclassified microorganisms of the Kairei and Pelagia areas

Due to the depth of sequencing provided by the current high-throughput sequencing technologies^85,86^, it is now routine to detect rare microbial taxa (relative abundance <0.1% of total community^85^). Rarity is likely a consequence of multiple environmental factors^18^. Previous studies have demonstrated that rare marine species exhibit different activity over time^87^: the rare biosphere in old hydrothermal chimneys can become dominant if environmental conditions change^88^ illustrating that the rare biosphere in a marine ecosystem is dynamic over time^89^.

Rare species among *Rhodobacterales* and *Rhizobiales* (*Alphaproteobacteria)* and *Desulfobacterales* (*Deltaproteobacteria*) and two rare species associated with the archaeal class *Thermoprotei* were identified “exclusively” in our chimney samples (Supplementary Table S7). These species are mainly involved in sulfur and metal cycling^36,90–92^. Given the prevalence of massive metal sulfides in the Kairei and Pelagia fields, the metabolisms of these rare species match local environmental features. Rare species from the bacterial class *Nitrospira*^93^ were also detected in our samples (Supplementary Table S7) and in other inactive vents in the southwest India Ridge^84^. They can perform complete denitrification and play an important role in nitrogen cycling^93^. Such rare biosphere is supposed to store ecological potential and facilitate a seed library of metabolisms. They may become active and predominant if environmental conditions become favorable with respect to their growth requirements^85,86^. Rare species are expected to be dormant, reducing their own activity/energy requirement, as a survival mechanism, to deal with the current unfavorable growth conditions^87^. They can be considered “losers” in the competition for nutrients during one certain time period^87^ and become “winners” at a later time point. Thus, species from the same class, like *Alphaproteobacteria*, *Deltaproteobacteria* and *Thermoprotei* in our study, could be predominant in some chimneys but could also be rare in other sites (Supplementary Tables S2–S4 and S7). Even if rare species are not active and/or low in abundance in inactive chimneys, mining of these and comparable habitats, i.e. SMS areas, may remove an important genetic reservoir from the deep-sea.

Our chimneys also appear to host large proportions of bacteria and archaea that are not detectable in any of the other investigated samples. The proportions of chimney *de novo* OTUs are much higher than those in most other hydrothermal vents (Supplementary Table S1). The unclassified species consist of “unknown” rare microbial species and other “unknown” organisms which have >0.1% abundance. Because these organisms are currently unculturable and their physiologies remain unknown, it is hard to predict the outcome of their possible disappearance due to deep-sea mining activities.

## Conclusions

The chimney communities, as analogues to SMS environments, appear to largely consist of habitat specific microorganisms including a large number of yet uncharacterized organisms. These unknown metabolisms make it the more difficult to predict what the impact will be if these organisms become unavailable for dispersal throughout the open ocean and to foresee the loss for contributing to local and possibly even distant biogeochemical cycling. Also striking is the high number of rare species present in these chimney environments. These habitats may represent a unique seed library for genetic resources. Removal of rare species in a given habitat may not appear crucial. However, as many of the rare sequences found in our analyses are very distinct from the known reference sequences, we only have a limited knowledge of the role they may play in other more growth-favorable environments, where they may be responsible for large element turnovers. Hence, the removal of SMS habitats through mining activities may not only have an impact on microbial diversity and biogeochemical cycling locally but may also be relevant on a global scale. In summary, it seems unjustifiable to remove a precious genetic resource from an unsustainable environment – for resolving current problems related to a high demand for minerals – given the limited understanding we have of the role that these organisms could play in the immediate or even in a distant environment.

## Material and Methods

### Samples, samples collection and sample processing

The fourteen samples (consisting of water, hydrothermal fluids, chimney structures or a sulfide talus sample) (see Table 2) were taken from the Kairei (25°S and 70°E) and Pelagia (26°S and 71°E) areas in the Indian Ocean during BGR’s INDEX2016 cruise with the *N/O Pourquoi pas?* (Ifremer, France). Hydrothermal fluids were collected with the KIPS-System^94^ mounted to the ROV VICTOR6000 (Ifremer). Seawater and plume samples were recovered with the tow-yo plume sled in SOPHI Niskin bottles at different depths. Chimney fragments and a massive sulfide block covered with a biofilm were sampled with the ROV and put into boxes (with a sealed lid) in order to avoid washing effects of the ambient seawater during subsequent sampling. Immediately after recovery of samples on board the water, plume and hydrothermal fluids were processed further and the chimney structures as well as the sulfide talus were stored at −80 °C. At least 500 ml of the open ocean water, plume and hydrothermal fluids were filtered each through 0.2 µm polycarbonate filters (type GTTP, Merck Millipore, Darmstadt, Germany). These were kept at −80 °C. For total cell counting, 37% formaldehyde was added to fluid samples to obtain a concentration of 4% formaldehyde for storage at 4 °C until microscopic analysis.

### DNA extraction, amplification and sequencing

For each chimney sample and the sulfide talus three 1 g rock subsamples were taken from the interior, the middle and the exterior (1 cm from the outer surface) parts of the chimney structure and homogenized using sterile mortar and pestles. DNA from filters, chimneys and sulfide talus was extracted using the PowerSoil^®^ DNA isolation Kit (MoBio, Carlsbad, CA, USA) according to the manufacturer’s instruction.

Bacterial 16S rRNA gene amplicons for the Illumina MiSeq system were prepared with the primers targeting the 16S V3 and V4 regions as previously described^33^. The archaeal 16S rRNA amplicons for the Illumina MiSeq system were prepared as the bacterial 16S rRNA amplicons. Primers used were forward primer: 5′-*TCGTCGGCAGCGTCAGATGTGTATAAGAGACAG* CAGCMGCCGCGGTAA-3′ and reverse primer: 5′-*GTCTCGTGGGCTCGGAGATGTGTATAAGAGACAG* GGCCATGCACCWCCTCTC-3′. The primers contain an Illumina adaptor overhang sequence (shown in italics) next to the broadly conserved primer sequence (S-D-Arch-0519-a-S-15/S-D-Arch-1041-a-A-18, shown in roman) as recommended by Illumina and a previous study^95^. Concentrations of all samples were estimated with a Qubit^®^ 2.0 Fluorometer (Thermo Fisher Scientific, Waltham, USA) and fragment lengths were analyzed by the 2100 Bioanalyzer using the DNA High Sensitivity Chip (Agilent Technologies, Santa Clara, USA). All samples were equally pooled and sequenced on the MiSeq platform (Illumina, St. Diego, USA).

### Sequence preprocessing

Quality control was performed after each step of preprocessing using Fastqc v.0.11.5 (http://www.bioinformatics.babraham.ac.uk/projects/fastqc/). As paired-end sequencing was used for the bacterial samples, we merged the reverse and forward read obtained from fragments shorter than the sum of the lengths of the two reads using Flash v.1.2.11^96^, with the following command line parameters: read length 251, average fragment length 460, standard deviation 46. Non-merging read pairs (3.77% of the read pairs) were thereby eliminated. The bacterial merged sequences and the archaeal reads (which were single-end) were quality-filtered, trimmed and formatted for further processing using the script split_libraries_fastq.py of Qiime v.1.9^97^, with default parameters.

### OTU picking

OTUs were picked from the sequences using the open-reference OTU picking procedure described in Rideout *et al*.^32^ and implemented in Qiime v.1.9^97^. Thereby, we employed the Silva database v.119^98^ and UCLUST v.1.2.22q^99^, using the default OTUs identity threshold level of 97%. OTUs consisting of a single sequence in the whole dataset were removed.

### Taxonomy and diversity analysis

Rarefaction curves were computed using the Qiime script alpha_rarefaction.py, using the number of OTUs as an estimation of the observed species. The beta diversity was computed using the Qiime script jackknifed_beta_diversity.py on a proportionally rescaled OTU table with an even count/site (10 million). Thereby the Sørensen-Dice and Bray-Curtis distances were used. Taxonomy plots were prepared based on the taxonomy assignments computed by the Qiime pipeline script pick_open_reference_otus.py.

### Publicly available data sets

The data for samples from Gonnella *et al*.^33^ was processed as described in the original manuscript. The data for samples 112 R, 115 R, 137, 138, 53 R and 55R^78^ were obtained from the archive http://jbpc.mbl.edu/research_supplements/g454/20060412-private/supplemental.zip. The data for samples FS312 and FS396^100^ was obtained from the Vamps database^101^, project KCK_SMT_Bv6. The data for sample GS08^80^ was obtained from the NCBI SRA database, runs SRR495221 and SRR495222. The data for samples Rb-1 to Rb-6 and LS-7 to LS-12^79^ were obtained from http://alrlab.research.pdx.edu/projects/MAR2008 (accessed in June 2012). The data for the archaeal samples Rb2A, Rb6A, Rb5A, LS7A, LS10, LS8A^79^ were obtained from the NCBI SRA database, experiment SRX03796. The data for samples TVG4 and TVG11^84^ were obtained from the NCBI SRA database, runs SRR2231135 and SRR2231136 (Bacteria) SRR2231031 and SRR2231032 (Archaea). The data for samples 3M23, 3M33, 7M24, 9M32, 9M4I, 9M4O and 9M4S^10^ were obtained from the Vamps database, project KCK_SBF. The data for samples VC and BP were obtained from the NCBI SRA runs associated with the NCBI BioProject PRJEB10564, and merging the samples VC1B, VC2B, VC4A, VC1A and VC2A (for VC) or BP1A, BP2B, BP3B, BP1B, BP3A, BP4A and BP2A (for BP)^102^. The data for samples F12-BP (Guaymas Basin), F12-LB (Lua Basin), F12-MH (Mariner hydrothermal vent field), F12-VF (Valu Fa Ridge), F12-LS (Lucky Strike vent field), F12-RB (Rainbow vent field), F12-TG (TAG vent field), were obtained from MG-Rast project mgp327, combining the 62 available samples by region^103^. The data for sample Nankai was obtained by combining the samples Nankai5cm and Nankai25cm, obtained from the NCBI SRA database, runs DRR001438 and DRR001439^83^.

OTUs were picked and taxonomy assigned to the data sets, each separately, by applying the same open-reference procedure described for our sequencing data. However, only reference-based OTUs were used for the meta-analysis, as the studies targeted different regions of the 16S rRNA genes.

### Cell numbers

For total cell counts, formaldehyde-fixed water samples were directly concentrated on filters. The cells on the filters were stained with SYBR Green I (Invitrogen) and embedded with moviol on black polycarbonate membrane filters (0.2 µm, Nuclepore, Whatman)^104^. The stained cells on the filters were counted by epifluorescence microscopy. Total cell counts were determined for the two open ocean seawater, plume and hydrothermal fluid samples.

### Sulfide precipitate characterization

During the INDEX2016 campaign hydrothermal precipitates were obtained using the remotely operated vehicle ROV VICTOR6000 owned by Ifremer. Ten exploring and sampling ROV dives were conducted at the Kairei and Pelagia hydrothermal vent fields recovering several active and inactive chimney edifices and fragments. Seven representative massive sulfide precipitates were chosen to analyze the mineralogical and geochemical composition of the inactive and actively venting chimneys with respect to the occurrence of bacterial communities (for sample description see Table 1). Hydrothermal precipitates are described and classified according to their morphology, macroscopic texture and mineralogy. The samples were prepared and analyzed for major element compositions by a combination of ICP emission and mass spectrometry at BGR and Actlabs, Ancaster, Ontario. Precision and accuracy were checked on replicates and against international standards (e.g. Oreas 75a, MP-1b, CZN-4, PTC-1b, and additional internationally certified standards). Polished sections were used for mineralogical and microanalytical characterization by optical and scanning electron microscopy and electron microprobe analyses, respectively.

### Data availability

Sequence data have been deposited in the National Center for Biotechnology Information Sequence Read Archive (SRA) under accession no. SRP120106.

## Electronic supplementary material

Supplementary material_Han_et_al

Supplementary Table S2: exclusive bacterial and archaeal OTUs present in the active or inactive chimneys.

Supplementary Table S3: exclusive bacterial and archaeal OTUs present in inactive chimneys versus all other samples.

Supplementary Table S4: exclusive bacterial and archaeal OTUs present in open ocean versus all chimneys samples.

Supplementary Table S5: significant differences between counts in distinct bacterial and archaeal groups found in relation to habitat types.

Supplementary Table S6: significant differences between counts in distinct bacterial and archaeal groups found in relation to mineralogy.

Supplementary table S7: rare species in Kairei and Pelagia fields.
