# Supplementary material for: Hydrothermal chimneys host habitat-specific microbial communities: analogues for studying the possible impact of mining seafloor massive sulfide deposits
Source: Sci Rep. 2018 Jul 10;8:10386. doi: 10.1038/s41598-018-28613-5 (PMC6039533; doi:10.1038/s41598-018-28613-5)
Supplement: Supplementary file 1 — Supplementary material_Han_et_al [file 41598_2018_28613_MOESM1_ESM.docx]

***Supplementary Material***

**Hydrothermal chimneys host habitat-specific microbial communities: analogues for studying the possible impact of mining seafloor massive sulfide deposits**

**Yuchen Han, Giorgio Gonnella, Nicole Adam, Axel Schippers, Lia Burkhardt, Stefan Kurtz, Ulrich Schwarz-Schampera, Henrike Franke, and Mirjam Perner^1^**

^1^Corresponding Author: Mirjam Perner. Mailing Address: GEOMAR Helmholtz Center for Ocean Research Kiel, Geomicrobiology, Wischhofstr. 1-3, 24148 Kiel. Tel: +49-431-600-2837, Fax: +49-40-431-600-2941, E-mail: mperner@geomar.de

**This file contains:**

**Supplementary Figures S1 to S4**

**Supplementary Table S1 to Table S7**

**Supplementary Figure S1:** Rarefaction curves of alpha diversity for Bacteria **(a)** and Archaea **(b)**. Abbreviations are as indicated in Table 2.

**
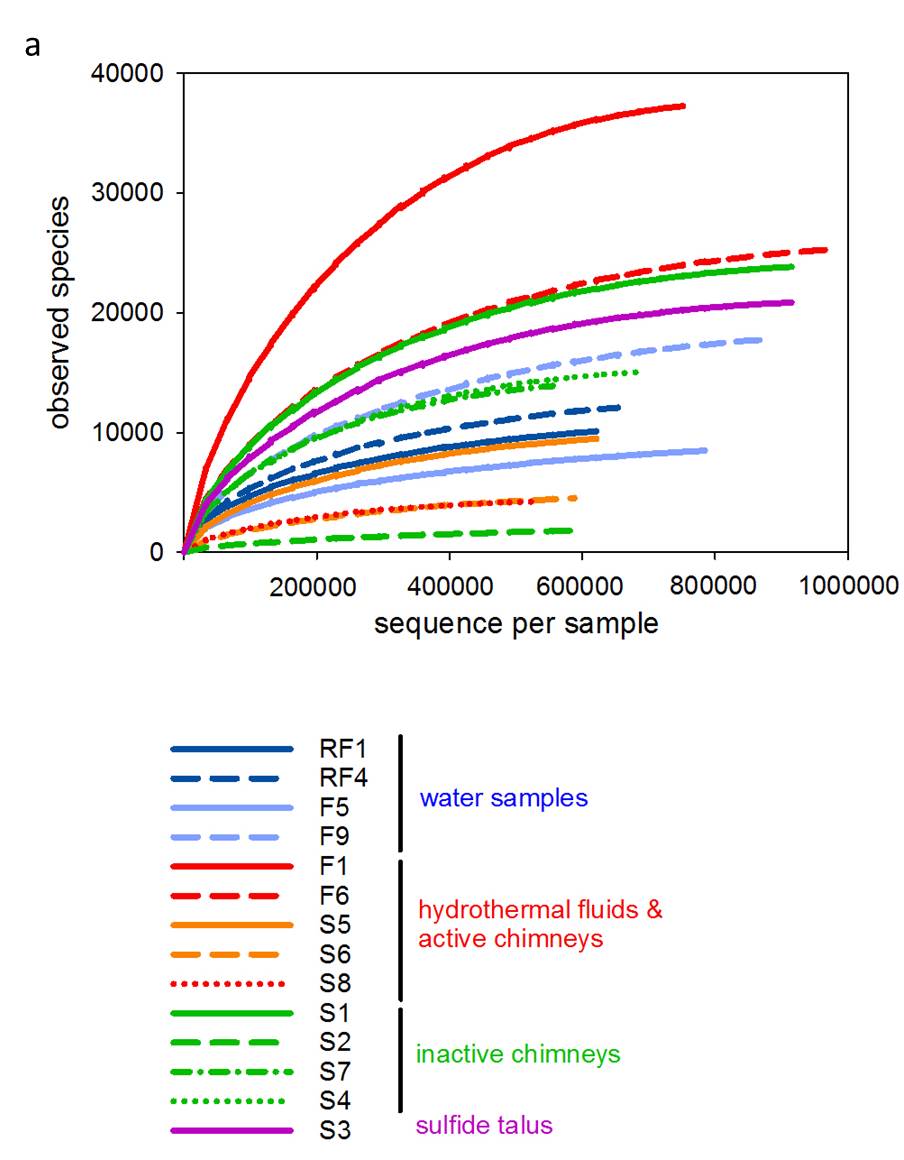
**

**b
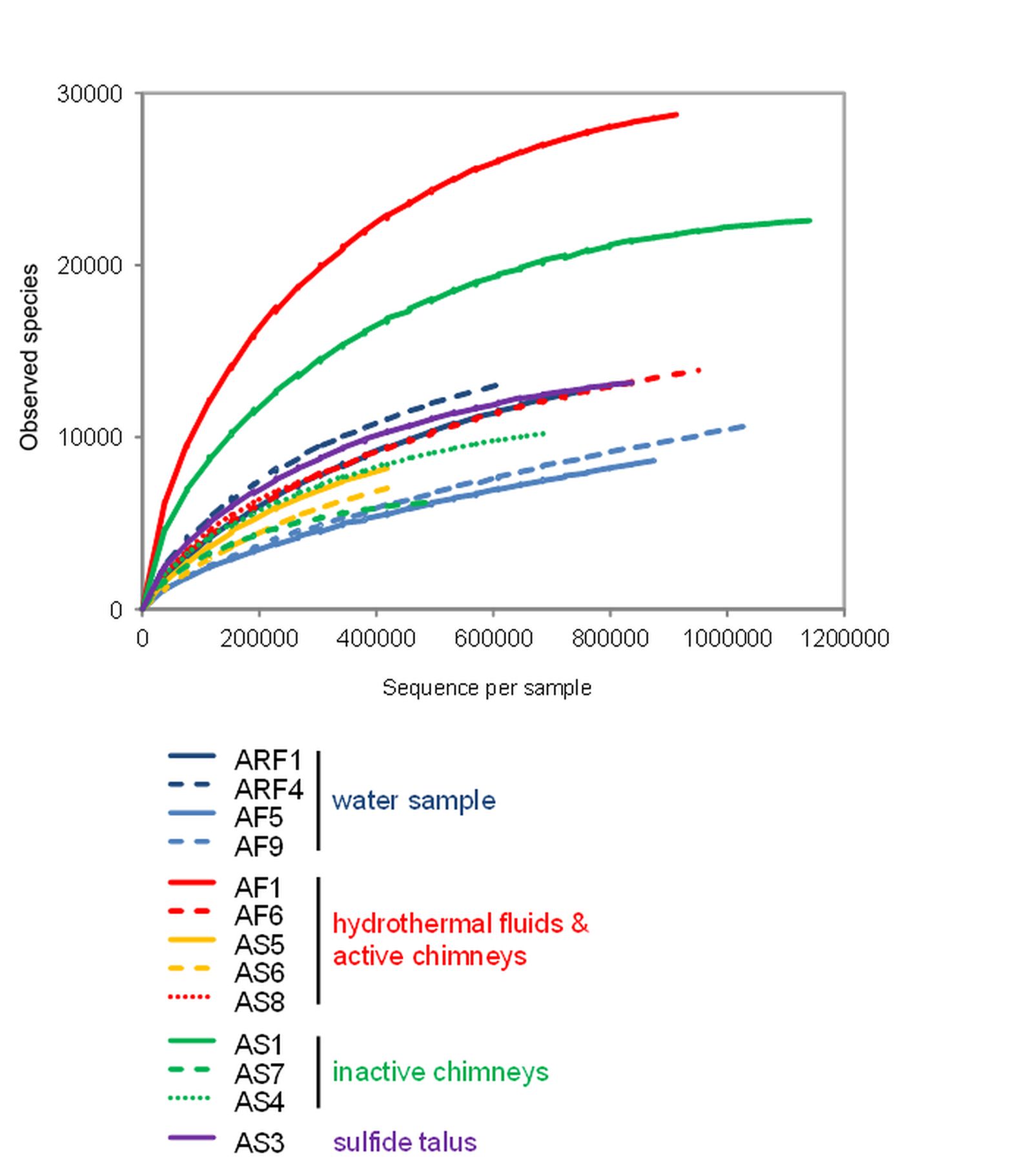
**

**Supplementary Figure S2:** Proportions of 16S tags related to major bacterial **(a, b, c)** and archaeal **(d, e, f)** phyla/class levels as indicated by reference-based and *de novo* **(a, d)**, only *de novo* **(b, e)** and only reference-based **(c, f)** sequences. For clarity, small groups, i.e. abundance less than 5%, were grouped.

**
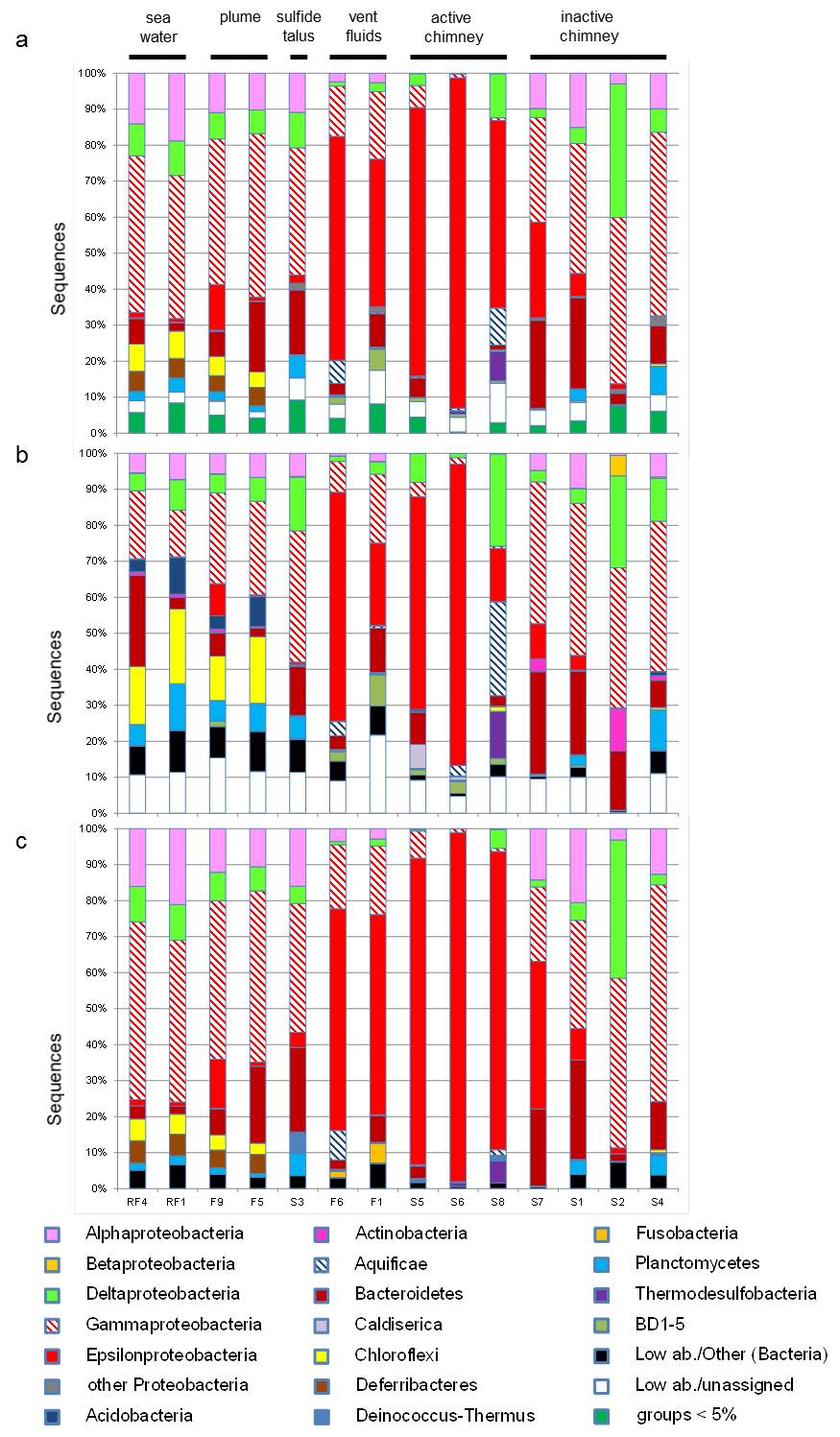
**

**
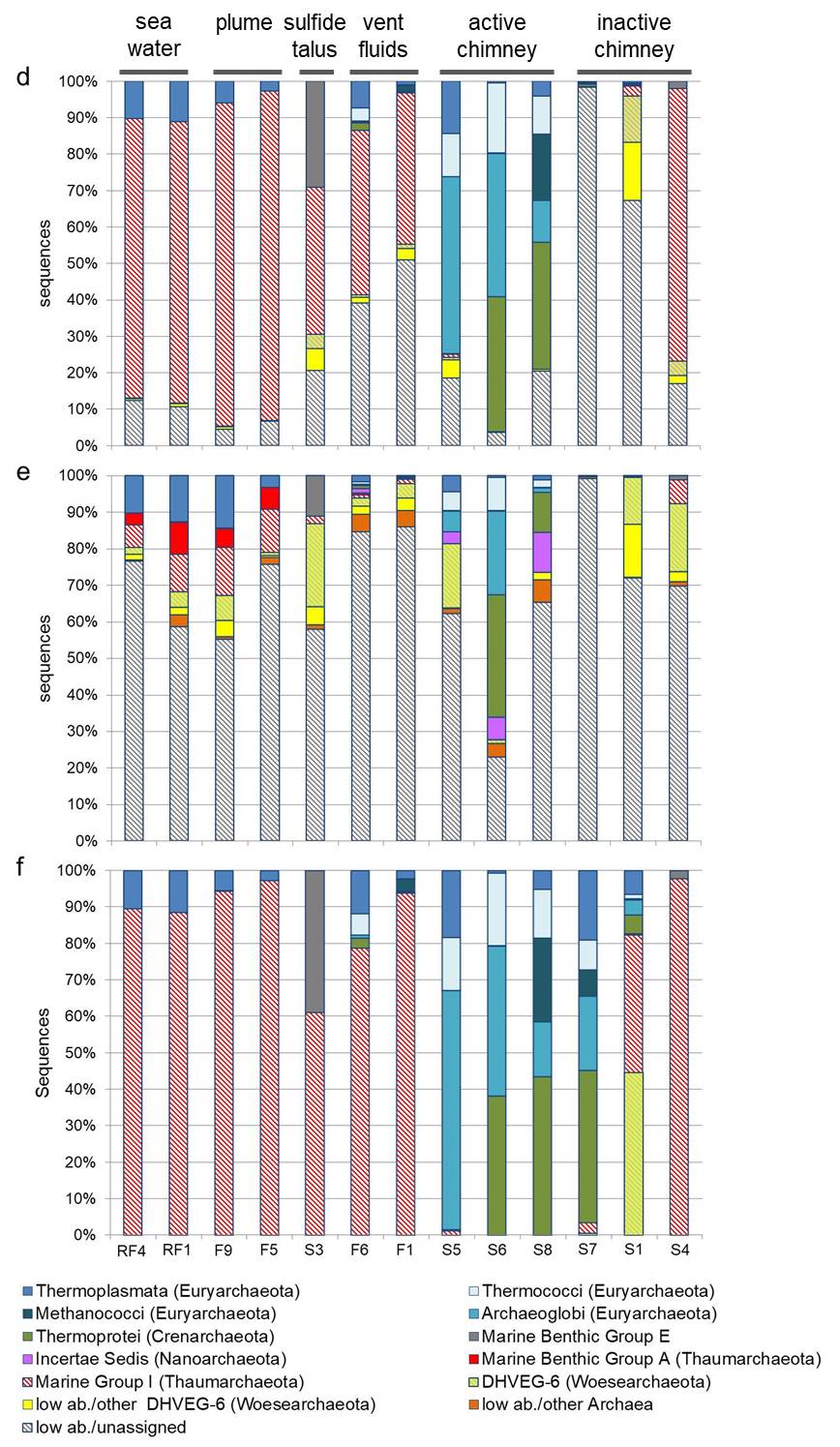
**

**Supplementary Figure S3:** Proportions of 16S tags related to bacterial orders as indicated by reference-based and *de novo* **(a)**, only *de novo* **(b)** and only reference-based **(c)** sequences. For clarity, small groups, i.e. abundance less than 10%, were grouped.

**
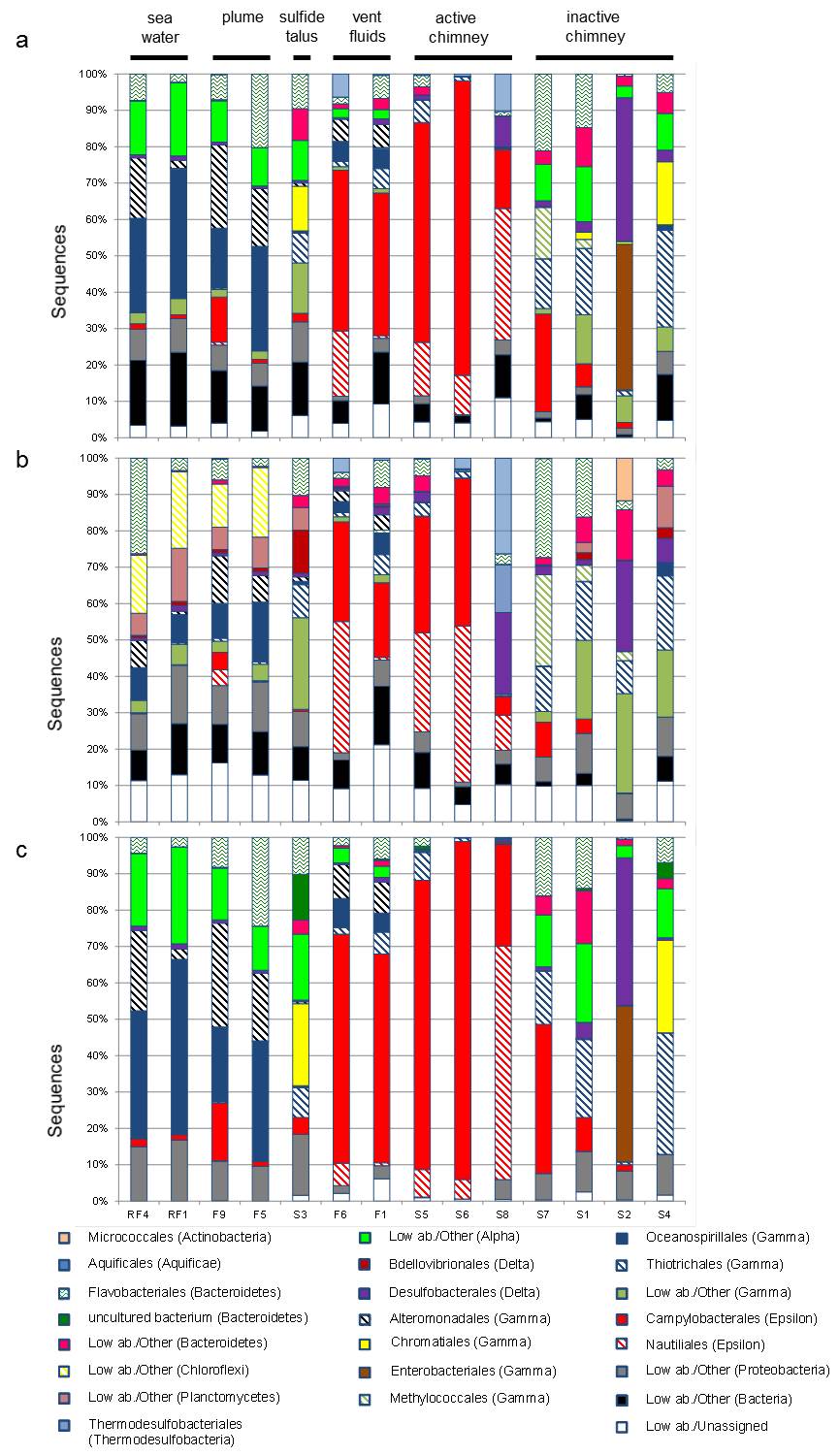
**

**Supplementary Figure S4:** Proportions of 16S tags related to bacterial **(a, b, c)** and archaeal **(d, e, f)** genera as indicated by reference-based and *de novo* **(a, d)**, only *de novo* **(b, e)** and only reference-based **(c, f)** sequences. For clarity, small bacterial and archaeal groups, i.e., abundance less than 10% and 5%, respectively, were grouped.

**
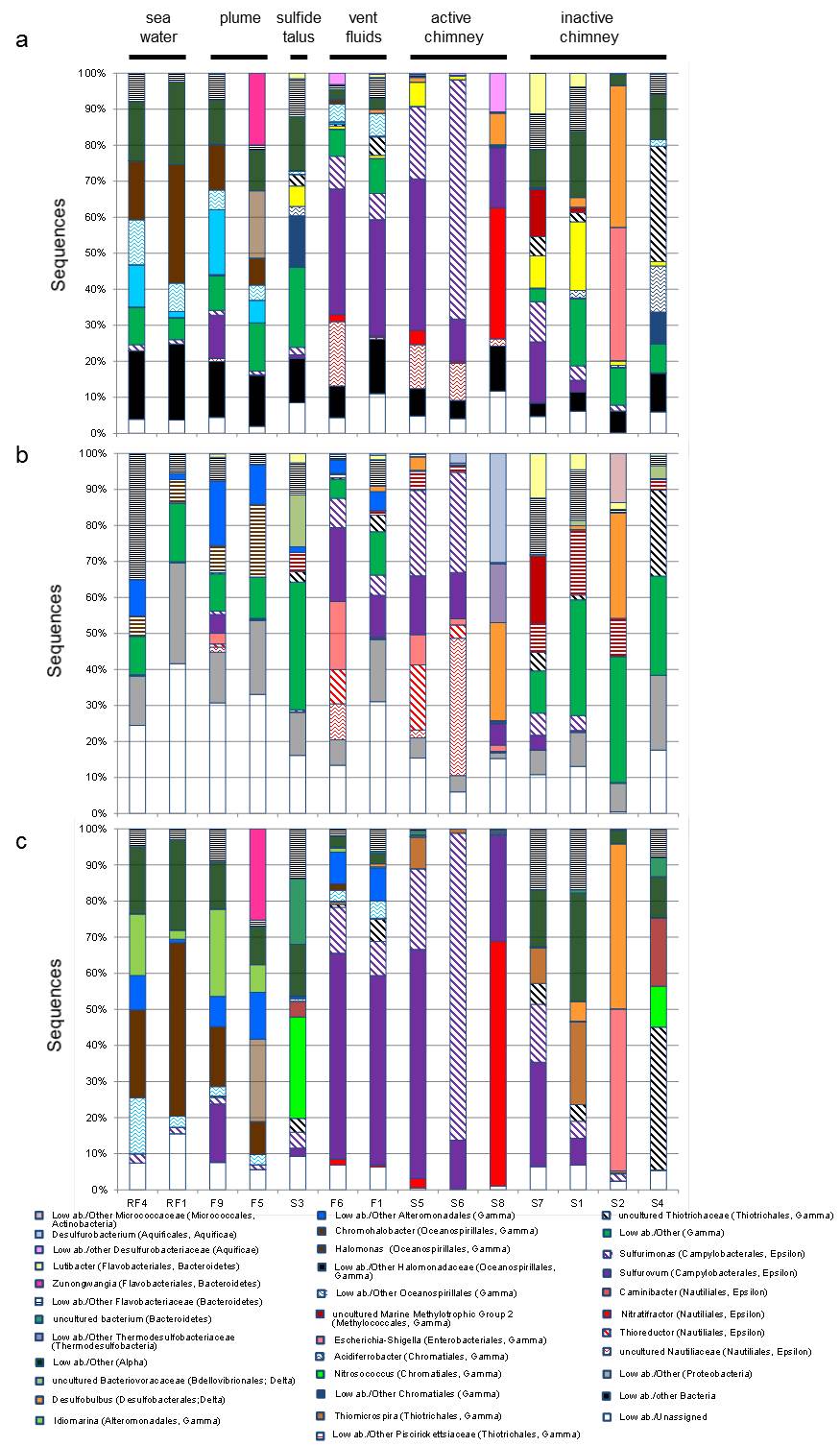
**

**
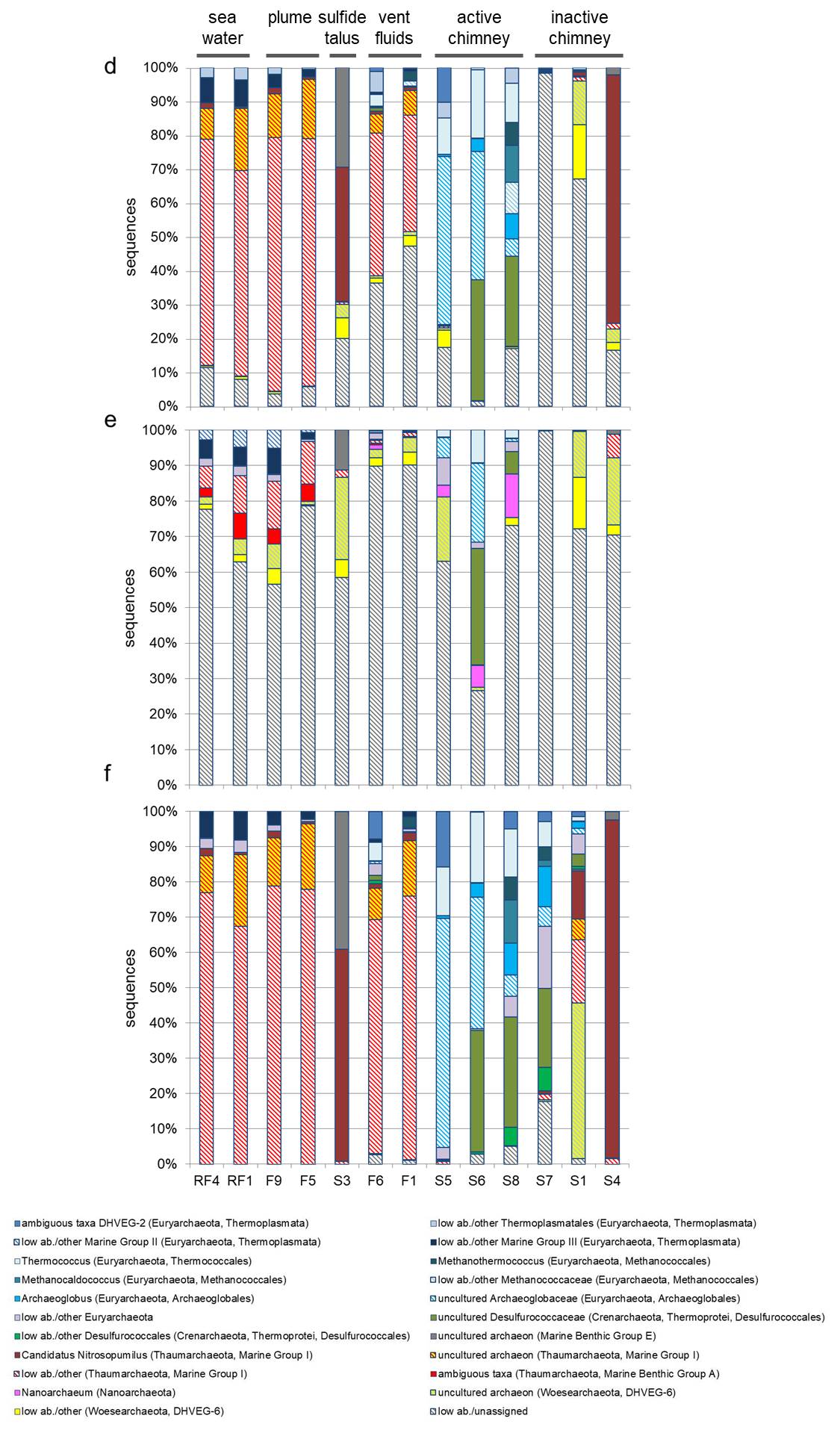
**

**Supplementary Table S1:** Counts, OTUs and proportions of *de novo* and reference-based sequences in this study and external studies.

| **This study** | ***de novo*** | | **reference-based** | |
| --- | --- | --- | --- | --- |
| **Bacteria** |  |  |  |  |
| **Site** | **count** | **proportions** | **count** | **proportions** |
| RF1 | 88234 | 13.96% | 543656 | 86.04% |
| RF4 | 111310 | 16.37% | 568608 | 83.63% |
| F5 | 68056 | 8.35% | 747241 | 91.65% |
| F1 | 326134 | 43.05% | 431484 | 56.95% |
| S8 | 196829 | 36.28% | 345651 | 63.72% |
| S1 | 461601 | 50.16% | 458657 | 49.84% |
| S2 | 42960 | 6.93% | 576511 | 93.07% |
| S7 | 262856 | 45.71% | 312239 | 54.29% |
| F9 | 142436 | 15.86% | 755411 | 84.14% |
| S3 | 468548 | 50.82% | 453337 | 49.18% |
| F6 | 399847 | 40.79% | 580422 | 59.21% |
| S5 | 235043 | 36.86% | 402554 | 63.14% |
| S6 | 90408 | 15.05% | 510487 | 84.95% |
| S4 | 298842 | 41.67% | 418353 | 58.33% |
|  |  |  |  |  |
| Total unique | 3193104 | 31.01% | 7104611 | 68.99% |
|  |  |  |  |  |
| **Site** | **OTUs** | **proportions** | **OTUs** | **proportions** |
| RF1 | 8104 | 79.82% | 2049 | 20.18% |
| RF4 | 10265 | 84.05% | 1948 | 15.95% |
| F5 | 6775 | 78.75% | 1828 | 21.25% |
| F1 | 34862 | 93.47% | 2436 | 6.53% |
| S8 | 3822 | 89.4% | 453 | 10.6% |
| S1 | 22481 | 94.2% | 1385 | 5.8% |
| S2 | 1145 | 61.2% | 726 | 38.8% |
| S7 | 13199 | 94.2% | 812 | 5.8% |
| F9 | 15406 | 86.11% | 2486 | 13.89% |
| S3 | 19505 | 93.38% | 1383 | 6.62% |
| F6 | 22960 | 90.63% | 2373 | 9.37% |
| S5 | 9006 | 94.24% | 550 | 5.76% |
| S6 | 4182 | 91.45% | 391 | 8.55% |
| S4 | 14143 | 93.37% | 1005 | 6.63% |
|  |  |  |  |  |
| Total unique | 134785 | 95.74% | 6000 | 4.26% |

| **This study** | ***de novo*** | | **reference-based** | |
| --- | --- | --- | --- | --- |
| **Archaea** |  |  |  |  |
| **Site** | **count** | **proportions** | **count** | **proportions** |
| ARF1 | 73824 | 9.63% | 692729 | 90.37% |
| ARF4 | 84974 | 13.25% | 556388 | 86.75% |
| AF5 | 49950 | 5.68% | 829840 | 94.32% |
| AF1 | 497021 | 52.86% | 443264 | 47.14% |
| AS8 | 74121 | 23.51% | 241167 | 76.49% |
| AS1 | 1049878 | 92.02% | 91048 | 7.98% |
| AS7 | 389496 | 77.21% | 114996 | 22.79% |
| AF9 | 53097 | 5.09% | 991020 | 94.91% |
| AS3 | 289600 | 33.94% | 563619 | 66.06% |
| AF6 | 374874 | 38.78% | 591874 | 61.22% |
| AS5 | 113750 | 27.08% | 306265 | 72.92% |
| AS6 | 23164 | 5.48% | 399421 | 94.52% |
| AS4 | 170510 | 24.45% | 526738 | 75.55% |
|  |  |  |  |  |
| Total unique | 3244259 | 33.82% | 6348369 | 66.18% |
|  |  |  |  |  |
| **Site** | **OTUs** | **proportions** | **OTUs** | **proportions** |
| ARF1 | 12393 | 96.65% | 429 | 3.35% |
| ARF4 | 12992 | 97.69% | 307 | 2.31% |
| AF5 | 8433 | 97.35% | 230 | 2.65% |
| AF1 | 28186 | 97.61% | 689 | 2.39% |
| AS8 | 7686 | 98.05% | 153 | 1.95% |
| AS1 | 22193 | 98.26% | 392 | 1.74% |
| AS7 | 6001 | 96.06% | 246 | 3.94% |
| AF9 | 10361 | 96.74% | 349 | 3.26% |
| AS3 | 12983 | 98.36% | 216 | 1.64% |
| AF6 | 13436 | 96.36% | 507 | 3.64% |
| AS5 | 8047 | 98.21% | 147 | 1.79% |
| AS6 | 6940 | 98.34% | 117 | 1.66% |
| AS4 | 10002 | 97.61% | 245 | 2.39% |
|  |  |  |  |  |
| Total unique | 119957 | 98.87% | 1376 | 1.13% |

Abbreviations as in Table 2, “A” means Archaea

| **Gonnella, et al. (2016)**[**^1^**](#_ENREF_1) | ***de novo*** | | **reference-based** | |
| --- | --- | --- | --- | --- |
| **Bacteria** |  |  |  |  |
| **Site** | **count** | **proportions** | **count** | **proportions** |
| WA1 | 444715 | 26.05% | 1262271 | 73.95% |
| WA2 | 115093 | 19.42% | 477527 | 80.58% |
| C | 166259 | 14.31% | 995934 | 85.69% |
| D | 109793 | 7.91% | 1278282 | 92.09% |
| FC | 83276 | 16.02% | 436552 | 83.98% |
| SP | 569573 | 33.42% | 1134894 | 66.58% |
| L1 | 1183014 | 38.61% | 1880926 | 61.39% |
| L2 | 846459 | 36.67% | 1462142 | 63.33% |
| L3 | 1470421 | 41.12% | 2105847 | 58.88% |
| L5 | 721424 | 26.03% | 2050228 | 73.97% |
| I2A | 68076 | 8.06% | 776489 | 91.94% |
| I2B | 316746 | 11.28% | 2490065 | 88.72% |
| I2C | 104758 | 9.33% | 1018476 | 90.67% |
| I2D | 351164 | 19.11% | 1486883 | 80.89% |
| I2E | 232352 | 11.65% | 1762379 | 88.35% |
| N | 195074 | 8.4% | 2126922 | 91.6% |
| OO | 1463501 | 18.52% | 6437940 | 81.48% |
|  |  |  |  |  |
| Total unique | 8441698 | 22.44% | 29183757 | 77.56% |
|  |  |  |  |  |
| **Site** | **OTUs** | **proportions** | **OTUs** | **proportions** |
| WA1 | 6153 | 86.54% | 957 | 13.46% |
| WA2 | 3030 | 76.23% | 945 | 23.77% |
| C | 5577 | 83.15% | 1130 | 16.85% |
| D | 5968 | 80.31% | 1463 | 19.69% |
| FC | 2908 | 73.64% | 1041 | 26.36% |
| SP | 5925 | 83.27% | 1190 | 16.73% |
| L1 | 17149 | 88.09% | 2318 | 11.91% |
| L2 | 16044 | 89.4% | 1902 | 10.6% |
| L3 | 17084 | 90.55% | 1783 | 9.45% |
| L5 | 17426 | 90.74% | 1778 | 9.26% |
| I2A | 2594 | 75.08% | 861 | 24.92% |
| I2B | 9811 | 80.74% | 2340 | 19.26% |
| I2C | 2934 | 74.32% | 1014 | 25.68% |
| I2D | 4739 | 79.12% | 1251 | 20.88% |
| I2E | 5937 | 78.13% | 1662 | 21.87% |
| N | 6357 | 79% | 1690 | 21% |
| OO | 13049 | 87.02% | 1947 | 12.98% |
|  |  |  |  |  |
| Total unique | 68081 | 92.37% | 5623 | 7.63% |

| **Flores, et al. (2011)** [**^2^**](#_ENREF_2) | ***de novo*** | | **reference-based** | |
| --- | --- | --- | --- | --- |
| **Bacteria** |  |  |  |  |
| **Site** | **count** | **proportions** | **count** | **proportions** |
| Rb-1 | 457 | 4.95% | 8770 | 95.05% |
| Rb-2 | 1818 | 40.82% | 2636 | 59.18% |
| Rb-4 | 447 | 4.61% | 9251 | 95.39% |
| Rb-5 | 304 | 2.9% | 10191 | 97.1% |
| Rb-6 | 3070 | 37.64% | 5087 | 62.36% |
| LS-10 | 983 | 18.14% | 4437 | 81.86% |
| Rb-3 | 2006 | 25% | 6017 | 75% |
| LS-7 | 1403 | 15.75% | 7507 | 84.25% |
| LS-8 | 2095 | 28.61% | 5227 | 71.39% |
| LS-9 | 1150 | 15.65% | 6200 | 84.35% |
| LS-11 | 1606 | 18.58% | 7037 | 81.42% |
| LS-12 | 1057 | 27.12% | 2841 | 72.88% |
|  |  |  |  |  |
| Total unique | 16396 | 17.90% | 75201 | 82.10% |
|  |  |  |  |  |
| **Site** | **OTUs** | **proportions** | **OTUs** | **proportions** |
| Rb-1 | 34 | 37.78% | 56 | 62.22% |
| Rb-2 | 78 | 51.66% | 73 | 48.34% |
| Rb-4 | 33 | 30.28% | 76 | 69.72% |
| Rb-5 | 45 | 25.14% | 134 | 74.86% |
| Rb-6 | 21 | 16.15% | 109 | 83.85% |
| LS-10 | 49 | 41.53% | 69 | 58.47% |
| Rb-3 | 114 | 47.7% | 125 | 52.3% |
| LS-7 | 125 | 56.31% | 97 | 43.69% |
| LS-8 | 116 | 47.15% | 130 | 52.85% |
| LS-9 | 41 | 32.8% | 84 | 67.2% |
| LS-11 | 41 | 44.57% | 51 | 55.43% |
| LS-12 | 31 | 26.96% | 84 | 73.04% |
|  |  |  |  |  |
| Total unique | 333 | 47.98% | 361 | 52.02% |

| **Sogin, et al. (2006)**[**^3^**](#_ENREF_3) | ***de novo*** | | **reference-based** | |
| --- | --- | --- | --- | --- |
| **Bacteria** |  |  |  |  |
| **Site** | **count** | **proportions** | **count** | **proportions** |
| 115R | 1457 | 9% | 14730 | 91% |
| 137 | 1588 | 11.48% | 12240 | 88.52% |
| 138 | 1336 | 10.31% | 11617 | 89.69% |
| 53R | 1450 | 11.41% | 11263 | 88.59% |
| 55R | 1455 | 14.96% | 8268 | 85.04% |
| 112R | 2590 | 17.07% | 12584 | 82.93% |
|  |  |  |  |  |
| Total unique | 9876 | 12.26% | 70702 | 87.74% |
|  |  |  |  |  |
| **Site** | **OTUs** | **proportions** | **OTUs** | **proportions** |
| 115R | 463 | 46.53% | 532 | 53.47% |
| 137 | 376 | 40.43% | 554 | 59.57% |
| 138 | 405 | 43.78% | 520 | 56.22% |
| 53R | 479 | 48.04% | 518 | 51.96% |
| 55R | 379 | 44.33% | 476 | 55.67% |
| 112R | 540 | 50.42% | 531 | 49.58% |
|  |  |  |  |  |
| Total unique | 1367 | 55.25% | 1107 | 44.75% |

| **Zhang, et al. (2016)**[**^4^**](#_ENREF_4) | ***de novo*** | | **reference-based** | |
| --- | --- | --- | --- | --- |
| **Bacteria** |  |  |  |  |
| **Site** | **count** | **proportions** | **count** | **proportions** |
| TVG4 | 2279 | 4.99% | 43392 | 95.01% |
| TVG11 | 2635 | 7.65% | 31814 | 92.35% |
|  |  |  |  |  |
| Total unique | 4914 | 6.13% | 75206 | 93.87% |
|  |  |  |  |  |
| **Site** | **OTUs** | **proportions** | **OTUs** | **proportions** |
| TVG4 | 569 | 34.63% | 1074 | 65.37% |
| TVG11 | 482 | 40.1% | 720 | 59.9% |
|  |  |  |  |  |
| Total unique | 676 | 37.66% | 1119 | 62.34% |

| **Huber, et al. (2007)**[**^5^**](#_ENREF_5) | ***de novo*** | | **reference-based** | |
| --- | --- | --- | --- | --- |
| **Universe** |  |  |  |  |
| **Site** | **count** | **proportions** | **count** | **proportions** |
| FS312 | 60143 | 13.76% | 376837 | 86.24% |
| FS396 | 93469 | 38.03% | 152324 | 61.97% |
|  |  |  |  |  |
| Total | 153612 | 22.50% | 529161 | 77.50% |
|  |  |  |  |  |
| **Site** | **OTUs** | **proportions** | **OTUs** | **proportions** |
| FS312 | 4144 | 67.99% | 1951 | 32.01% |
| FS396 | 1729 | 61.68% | 1074 | 38.32% |
|  |  |  |  |  |
| Total unique | 4967 | 70.31% | 2097 | 29.69% |

| **Jaeschke, et al. (2012)**[**^6^**](#_ENREF_6) | ***de novo*** | | **reference-based** | |
| --- | --- | --- | --- | --- |
| **Universe** |  |  |  |  |
| **Site** | **count** | **proportions** | **count** | **proportions** |
| GS08 | 2007 | 47.49% | 2219 | 52.51% |
|  |  |  |  |  |
| **Site** | **OTUs** | **proportions** | **OTUs** | **proportions** |
| GS08 | 47 | 58.02% | 34 | 41.98% |

| **Flores, et al. (2011)** [**^2^**](#_ENREF_2) | ***de novo*** | | **reference-based** | |
| --- | --- | --- | --- | --- |
| **Archaea** |  |  |  |  |
| **Site** | **count** | **proportions** | **count** | **proportions** |
| Rb5A | 291 | 6.17% | 4426 | 93.83% |
| LS10 | 1117 | 78.22% | 311 | 21.78% |
| LS7A | 2236 | 58.58% | 1581 | 41.42% |
| LS8A | 870 | 65.17% | 465 | 34.83% |
| Rb2A | 3117 | 57.72% | 2283 | 42.28% |
| Rb6A | 2382 | 47.39% | 2644 | 52.61% |
| LS11 | 2592 | 54.66% | 2150 | 45.34% |
|  |  |  |  |  |
| Total unique | 12605 | 47.63% | 13860 | 52.37% |
|  |  |  |  |  |
| **Site** | **OTUs** | **proportions** | **OTUs** | **proportions** |
| Rb5A | 39 | 40.62% | 57 | 59.38% |
| LS10 | 43 | 53.75% | 37 | 46.25% |
| LS7A | 140 | 66.35% | 71 | 33.65% |
| LS8A | 85 | 61.15% | 54 | 38.85% |
| Rb2A | 117 | 62.23% | 71 | 37.77% |
| Rb6A | 34 | 44.16% | 43 | 55.84% |
| LS11 | 59 | 55.14% | 48 | 44.86% |
|  |  |  |  |  |
| Total unique | 276 | 60.93% | 177 | 39.07% |

| **Flores, et al. (2012)** [**^7^**](#_ENREF_7) | ***de novo*** | | **reference-based** | |
| --- | --- | --- | --- | --- |
| **Archaea** |  |  |  |  |
| **Site** | **count** | **proportions** | **count** | **proportions** |
| F12-TG | 1975 | 13.67% | 12475 | 86.33% |
| F12-LB | 16501 | 19.38% | 68644 | 80.62% |
| F12-GB | 13583 | 24.07% | 42854 | 75.93% |
| F12-LS | 1766 | 10.64% | 14830 | 89.36% |
| F12-RB | 3200 | 11.22% | 25315 | 88.78% |
| F12-VF | 1994 | 16.48% | 10109 | 83.52% |
| F12-MH | 4025 | 20.17% | 15929 | 79.83% |
|  |  |  |  |  |
| Total unique | 43044 | 18.46% | 190156 | 81.54% |
|  |  |  |  |  |
| **Site** | **OTUs** | **proportions** | **OTUs** | **proportions** |
| F12-TG | 70 | 55.12% | 57 | 44.88% |
| F12-LB | 456 | 68.26% | 212 | 31.74% |
| F12-GB | 488 | 70.42% | 205 | 29.58% |
| F12-LS | 106 | 54.08% | 90 | 45.92% |
| F12-RB | 108 | 52.17% | 99 | 47.83% |
| F12-VF | 107 | 60.11% | 71 | 39.89% |
| F12-MH | 149 | 54.18% | 126 | 45.82% |
|  |  |  |  |  |
| Total unique | 888 | 74.25% | 308 | 25.75% |

| **Zhang, et al. (2016)**[**^4^**](#_ENREF_4) | ***de novo*** | | **reference-based** | |
| --- | --- | --- | --- | --- |
| **Archaea** |  |  |  |  |
| **Site** | **count** | **proportions** | **count** | **proportions** |
| (A)TVG11 | 8468 | 10.31% | 73657 | 89.69% |
| (A)TVG4 | 47024 | 26.48% | 130548 | 73.52% |
|  |  |  |  |  |
| Total unique | 55492 | 21.37% | 204205 | 78.63% |
|  |  |  |  |  |
| **Site** | **OTUs** | **proportions** | **OTUs** | **proportions** |
| (A)TVG11 | 624 | 82.21% | 135 | 17.79% |
| (A)TVG4 | 891 | 91.86% | 79 | 8.14% |
|  |  |  |  |  |
| Total unique | 1283 | 89.91% | 144 | 10.09% |

**Supplementary Table S2:** exclusive bacterial and archaeal OTUs present in active versus inactive chimneys.

**Supplementary Table S3:** exclusive bacterial and archaeal OTUs present in inactive chimneys versus all other samples.

**Supplementary Table S4:** exclusive bacterial and archaeal OTUs present in open ocean versus all chimneys samples.

**Supplementary Table S5:** significant differences between counts in distinct bacterial and archaeal groups found in relation to habitat types.

**Supplementary Table S6:** significant differences between counts in distinct bacterial and archaeal groups found in relation to mineralogy.

**Supplementary Table S7:** rare species in Kairei and Pelagia fields.

**References:**

1 Gonnella, G. *et al.* Endemic hydrothermal vent species identified in the open ocean seed bank. *Nat. Microbiol.* **1**, doi: 10.1038/nmicrobiol.2016.1086, (2016).

2 Flores, G. E. *et al.* Microbial community structure of hydrothermal deposits from geochemically different vent fields along the Mid-Atlantic Ridge. *Environ. Microbiol.* **13**, 2158-2171, (2011).

3 Sogin, M. L. *et al.* Microbial diversity in the deep sea and the underexplored “rare biosphere”. *Proc. Natl. Acad. Sci. U.S.A.* **103**, 12115-12120, (2006).

4 Zhang, L. *et al.* Bacterial and archaeal communities in the deep-sea sediments of inactive hydrothermal vents in the Southwest India Ridge. *Sci. Rep.* **6**, doi: 10.1038/srep25982, (2016).

5 Huber, J. A. *et al.* Microbial population structures in the deep marine biosphere. *Science* **318**, 97-100, (2007).

6 Jaeschke, A. *et al.* Microbial diversity of Loki's Castle black smokers at the Arctic Mid-Ocean Ridge. *Geobiology* **10**, 548-561, (2012).

7 Flores, G. E., Wagner, I. D., Liu, Y. & Reysenbach, A.-L. Distribution, abundance, and diversity patterns of the thermoacidophilic “deep-sea hydrothermal vent euryarchaeota 2”. *Front. Microbiol.* **3**, 47, (2012).
